# A Review on Cu_2_O-Based Composites in Photocatalysis: Synthesis, Modification, and Applications

**DOI:** 10.3390/molecules28145576

**Published:** 2023-07-22

**Authors:** Qian Su, Cheng Zuo, Meifang Liu, Xishi Tai

**Affiliations:** College of Chemistry & Chemical and Environmental Engineering, Weifang University, Weifang 261061, China; sqian316@wfu.edu.cn

**Keywords:** heterojunction, photocatalysis, synthesis, modification, application

## Abstract

Photocatalysis technology has the advantages of being green, clean, and environmentally friendly, and has been widely used in CO_2_ reduction, hydrolytic hydrogen production, and the degradation of pollutants in water. Cu_2_O has the advantages of abundant reserves, a low cost, and environmental friendliness. Based on the narrow bandgap and strong visible light absorption ability of Cu_2_O, Cu_2_O-based composite materials show infinite development potential in photocatalysis. However, in practical large-scale applications, Cu_2_O-based composites still pose some urgent problems that need to be solved, such as the high composite rate of photogenerated carriers, and poor photocatalytic activity. This paper introduces a series of Cu_2_O-based composites, based on recent reports, including pure Cu_2_O and Cu_2_O hybrid materials. The modification strategies of photocatalysts, critical physical and chemical parameters of photocatalytic reactions, and the mechanism for the synergistic improvement of photocatalytic performance are investigated and explored. In addition, the application and photocatalytic performance of Cu_2_O-based photocatalysts in CO_2_ photoreduction, hydrogen production, and water pollution treatment are discussed and evaluated. Finally, the current challenges and development prospects are pointed out, to provide guidance in applying Cu_2_O-based catalysts in renewable energy utilization and environmental protection.

## 1. Introduction

With the development of industrialization, the use of fossil fuels in industry has caused many problems, such as carbon dioxide emissions causing global warming, water pollution, and the destruction of surrounding biological habitats. Energy shortages and environmental pollution pose a serious threat to the development of industry and agriculture, and have become hot topics that need to be addressed [1,2]. Photocatalytic technology utilizes semiconductor materials to achieve the photoreduction of CO_2_, the photocatalytic decomposition of water, and the degradation of pollutants, and has the advantages of a low cost, simple operation, and no secondary pollution [3,4,5]. Photocatalysis is a green technology that fully utilizes solar energy, and is considered one of the most feasible and promising methods to solve environmental and energy problems.

Since Fukushima et al. [6] discovered in 1967 that TiO_2_ can decompose water to produce hydrogen under light, tremendous progress has been made in photocatalytic technology. Due to its stable structure, high efficiency, low cost, nontoxicity, and high optical stability, TiO_2_ has become widely studied in the past few decades [7,8]. However, TiO_2_ can only absorb 3–5% of total ultraviolet light, so its utilization of sunlight is not high, significantly limiting its practical application under sunlight [9,10]. To effectively utilize the maximum proportion of visible light covering the solar spectrum (λ > 400 nm), in addition to modifying TiO_2_, researchers have studied a series of novel photocatalysts with a visible light response, such as simple oxides (ZnO [11] and Cu_2_O [12]), sulfides (CdS [13] and MoS_2_ [14]), Bi-based materials (Bi_2_WO_6_ [15] and BiVO_4_ [16]), and nitrides (C_3_N_4_ [17]).

Compared with other semiconductors, copper(I) oxide (Cu_2_O) has the advantages of nontoxicity, a favorable environmental acceptability, low cost, and high activity. It has been widely used in solar cells [18], carbon monoxide oxidation [19], photocatalysts [20], electrocatalysts [21], and sensors [22]. As a p-type semiconductor, Cu_2_O has a bandgap width of 2.17 eV, and a broad response range to the solar spectrum. A Cu_2_O material is currently one of the most promising visible light photocatalysts, and has become a research hotspot in photocatalysis. Li et al. [23] researched cubic c-Cu_2_O with the main exposure surface of (100), and tested its photocatalytic degradation performance on methyl orange (MO). It could completely decompose MO in an aqueous solution within 80 min under visible light, and almost remained unchanged in five consecutive cycles, showing a satisfactory stability. However, the application prospects of Cu_2_O in photocatalysis have been limited due to its poor stability, susceptibility to photocorrosion, and low quantum yield. To further improve the photocatalytic performance and stability of Cu_2_O, researchers have focused on a series of studies on morphology control, heteroatom doping, and the construction of semiconductor heterojunctions. With the increasing maturity in preparation and detection methods, the research on improving the photocatalytic activity of Cu_2_O has become more in-depth and diversified.

Unlike other reports in the literature [24,25,26], this paper reviews the preparation methods and applications of different Cu_2_O-based composites reported in recent years. By analyzing the roles of the different components in the photocatalytic process, we explain the reasons behind the improvement of photocatalytic performance, and point out the future direction that the industrial application of Cu_2_O-based composites could take.

**Figure 1:**
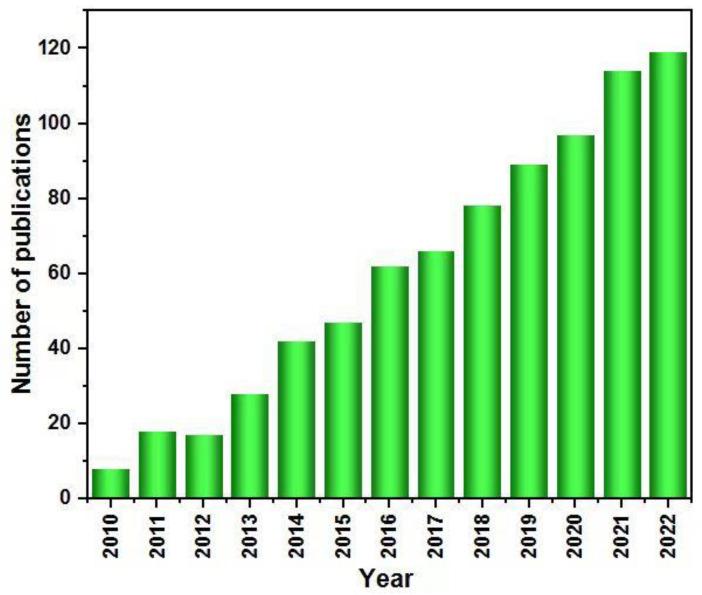
The annual number of publications using “Cu_2_O” as a topic keyword since 2010 (data taken from Web of Science on 1 January 2023).

**Figure 2:**
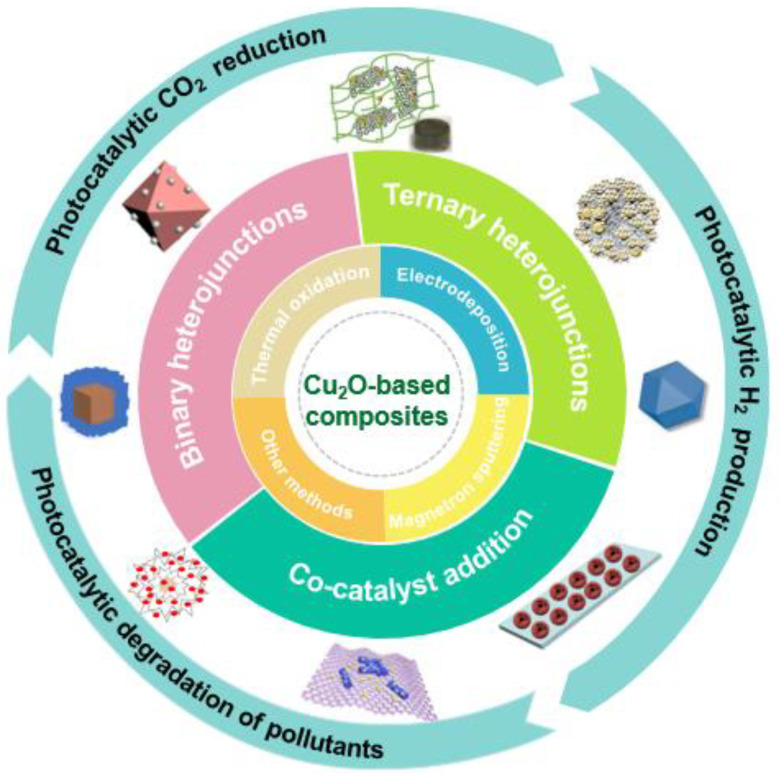
The research framework and basis of thinking for this review.

According to data from the Web of Science platform by Clarivate Analytics (Figure 1), research on the subject of the photocatalysis of Cu_2_O and Cu_2_O-based materials is increasing year by year, indicating that Cu_2_O-based materials are becoming ideal candidates for a variety of energy and environmental photocatalysis applications. Based on the research direction of Cu_2_O photocatalysts, in this paper, the main preparation methods are introduced, including their merits and demerits, and the current main research focus and progress are reviewed. The research ideas and framework of this review are shown in Figure 2. The frequently used methods for improving their photocatalytic performance are reviewed, including morphology and size improvement, doping, metal loading, and semiconductor hybridization. In addition, several representative Cu_2_O-based composite photocatalysts are introduced, including metal/Cu_2_O composite, Cu_2_O semiconductor composite, Cu_2_O/carbon material composite, and ternary composite photocatalysts. Their applications in the photocatalytic reduction of CO_2_, photocatalytic hydrolysis of hydrogen, photocatalytic degradation of pollutants, and photocatalytic reduction of metal atoms are discussed through the combination of the experimental numbers and reaction principles. Finally, the future development of Cu_2_O-based composite photocatalysts is considered.

## 2. Synthetic Methods

The low utilization of visible light by single CuO materials, and the easy complexation of electron–hole pairs generated by CuO under photoexcitation limit the application of CuO materials in photocatalysis. There is currently more research on doping Cu_2_O with high Li or Na metal concentrations. The bonding network of the off-domain two- and three-electron centers is disrupted, effectively localizing the electrons in the limited space.

### 2.1. Preparation Methods

**Figure 3:**
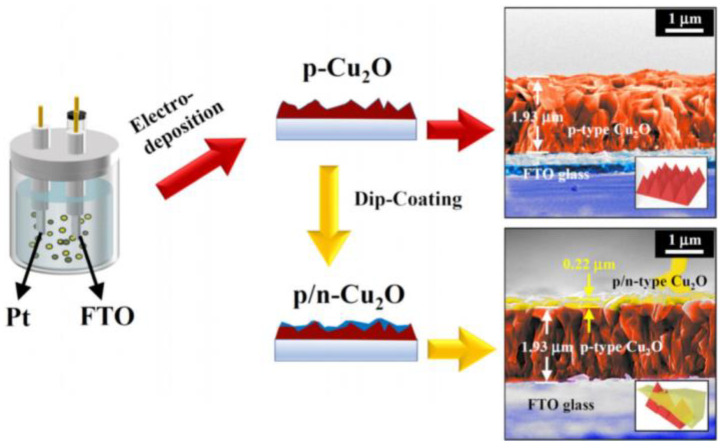
Schematic diagram of the procedure for the electrodeposition of p/n Cu_2_O on the FTO substrate [28].

Different preparation methods to prepare effective Cu_2_O materials for photocatalytic experiments have been reported in the literature. The thermal oxidation of metals is a widely used method for synthesizing high-quality oxides. The final desired thickness of the Cu_2_O layer is prepared based on the oxidation of the high-purity copper foil. The temperature range is between 1000 and 1500 °C under a pure oxygen atmosphere or mixed gas atmosphere (e.g., Ar + O_2_). The obtained Cu_2_O is polycrystalline, with different grain structures depending on the chosen experimental conditions. In general, a mixture of CuO and Cu_2_O appears in copper foil at the end of oxidation. The Cu_2_O appears first, at the beginning of the oxidation process, while the CuO takes a long time to appear in the oxidation process. Electrodeposition is one of the methods for the production of high-quality Cu_2_O. The advantages of this method are that it is cheap, can efficiently work on different substrates, and allows for adjustments in the material properties and morphology according to the following parameters: the applied potential, current, and temperature, and the pH of the tank solution. The first electrochemical synthesis of Cu_2_O was by Stareck [27]. Subsequently, many other scholars developed different synthesis methods, using copper precursors, electrolytes, and electrochemistry. Two types of Cu_2_O nanoparticles were successfully synthesized by adjusting the pH of the electrolyte [28]. Firstly, the pH of the electrolyte was adjusted to 10, and a pyramid-shaped p-type Cu_2_O crystal was grown on the FTO substrate. Subsequently, the pH was adjusted to 4.9, and an ultra-thin layer of n-Cu_2_O deposition product was obtained (Figure 3). The p-Cu_2_O nanoparticles and the n-Cu_2_O protective layer on the surface formed a p/n heterojunction. The modified p/n-Cu_2_O had a bandgap of 2–2.2 eV, and could be excited by visible light with a wavelength less than 600 nm. Its photocurrent response was significantly improved, increasing the charge transfer rate and the stability of the catalyst. Thus, the modified p/n-Cu_2_O catalyst exhibited much higher activity than the original p-Cu_2_O.

**Figure 4:**
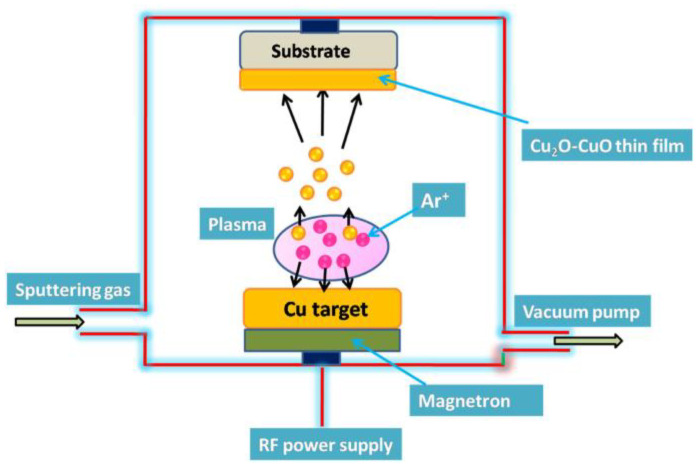
Schematic diagram of a Cu_2_O–CuO film prepared using the magnetron sputtering method [29]. Copyright 2020, Elsevier.

Magnetron sputtering is a process that uses high-energy particles to bombard a solid target, so that atoms or molecules sputtered from the surface of the target form a thin film in a specific region. The CuO films prepared using this method exhibit nanometer-sized columnar structures, and the crystallinity, grain size, and film thickness of the Cu_2_O films can be controlled by varying the sputtering parameters (e.g., the sputtering power, oxygen content, oxygen concentration, sputter deposition time, and annealing temperature). Cu_2_O–CuO films with an excellent photocatalytic performance have been deposited on glass substrates using RF magnetron sputtering (Figure 4) [29]. It has been observed that with the prolongation of the sputtering deposition time, the size of the Cu_2_O–CuO nanoparticles has increased from 7 nm to 13 nm, and the thickness of the thin films from 7 nm to 50 nm, resulting in a rougher surface, reduced bandgap, and decreased PL strength. The results indicate that the structure, morphology, and optical and photocatalytic properties of prepared Cu_2_O–CuO films are strongly dependent on the deposition thickness. Under sunlight exposure, Cu_2_O–CuO films can completely degrade pollutants (methylene blue and methyl orange) from water within only 60 min.

### 2.2. Other Methods

**Figure 5:**
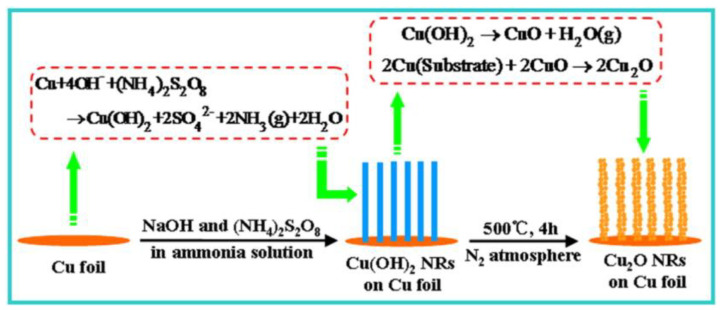
The schematic illustration of the synthesis process of Cu_2_O nanorod array films [42]. Copyright 2016, Elsevier.

In addition to the above methods, different surfactants [30,31,32,33,34,35] and micelles [36] have been used, mainly to control the morphology of the prepared Cu-based catalyst particles. Cu_2_O nanocrystals and nanoarray with cubic [37,38,39], octahedral [40], and multipod structures [41] have been prepared using these methods. Yang et al. [42] have proposed a metal-induced thermal reduction (MITR) method for the in-situ growth of Cu_2_O crystals on a copper substrate. The corresponding scheme is shown in Figure 5, and the operation is divided into two steps: (a) under alkaline conditions, the Cu(OH)_2_ nanorod array is in-situ grown by impregnating copper foil with a mixed solution of (NH_4_)_2_S_2_O_8_ and ammonia; and (b) the Cu(OH)_2_ on copper foil is directly thermally reduced to Cu_2_O nanorod array films in a N_2_ atmosphere at 500 °C. The average diameter of a nanorod was 400 ± 100 nm, with a length of several micrometers. The method is simple and efficient, and the preparation process has a low energy consumption and is controllable. In addition, the introduction of the substrate metal Cu can significantly reduce the reduction temperature, by changing the Gibbs free energy of the reaction. Surfactant-free synthesis has also been developed to reduce the interference of these surfactants [43,44,45]. Solvothermal [46,47] and sol–gel [48] methods have also been tested. The wet chemistry route [49,50], thermal evaporation [51,52], chemical vapor deposition [53,54], and hydrothermal route [55,56,57,58] are also common methods for synthesizing such semiconductors. In addition, the corresponding properties of Cu_2_O-based materials synthesized by different methods are detailed in the Section 4, including their morphology, structure, band gap, and photocatalytic applications.

## 3. Modification Strategies

Although noble metals have been used in photocatalytic organic waste degradation and CO_2_ reduction, their efficiency is still high. However, the cost is also high (e.g., Pt and Au), making them unsuitable for future industrial development. In contrast, Cu_2_O is inexpensive to use. It also has excellent CO_2_ capture ability and photochemical and structural properties, and shows unlimited development potential in CO_2_ reduction. However, the high electron–hole complexation rate and the low optical quantum efficiency limit the application of Cu_2_O in photocatalysis. To improve the photocatalytic efficiency of Cu_2_O, the structure of Cu_2_O needs to be modified. The modified structures are mainly divided into binary and ternary Cu_2_O heterostructure structures, and the addition of co-catalysts, in this section.

### 3.1. Binary Cu_2_O-Based Heterojunctions

#### 3.1.1. Cu_2_O/Noble Metal Heterojunction

The Fermi energy level of the noble metal material is relatively low compared to that of the catalyst in the photocatalytic reduction of CO_2_, which has a higher work function than that of the catalyst. The mutual contact between the two will form a Schottky barrier at the metal–semiconductor interface, which can effectively inhibit the complexation of photoexcited electron–hole pairs, thus promoting the catalytic process, and improving the catalytic efficiency of the catalyst. The currently synthesized Cu_2_O/noble metal composites are Cu_2_O/Ag [59], Cu_2_O/Au [60], and Cu_2_O/Pt [61]. These materials show more than 90% photocatalytic efficiency for modified Cu_2_O.

Cu_2_O/Au nanostructures have been extensively investigated in recent years. Kuo et al. [62] reported the synthesis of Au@Cu_2_O core–shell nanocrystals using a chemical reduction method. The nanocrystals exhibited high activity in degrading methyl orange. Ag is relatively inexpensive, and has a higher electron transfer efficiency than metallic Au. Therefore, Ag/Cu_2_O catalysts have been more widely studied. Yang et al. [63] prepared Cu_2_O/Ag spherical microstructures by depositing silver nanoparticles on the surface of Cu_2_O through the thermal decomposition of silver acetate.

#### 3.1.2. Cu_2_O/Graphene (GO) Heterojunction

From amorphous carbon black to crystalline structured natural layered graphite, and from zero-dimensional nanostructured fullerenes to two-dimensional structured graphene, carbon materials have been the most widely used and endlessly promising materials on earth. In recent decades, carbon nanomaterials have attracted much attention. The discovery of graphene self-assembled hydrogels with three-dimensional mesh structures has dramatically enriched the carbon material family, and provided a new growth point for new materials. Due to their unique nanostructure and properties, they have also shown significant scientific significance and experimental results. Thus, they provide a new target and direction when it comes to researching carbon-based materials. Graphene has been compounded with semiconductor photocatalysts, using its regular two-dimensional planar structure as a photocatalyst carrier. On the one hand, this could improve the dispersion of the catalyst. On the other hand, it could accelerate the photogenerated charge migration rate, and improve the photocatalytic activity of the composites.

**Figure 6:**
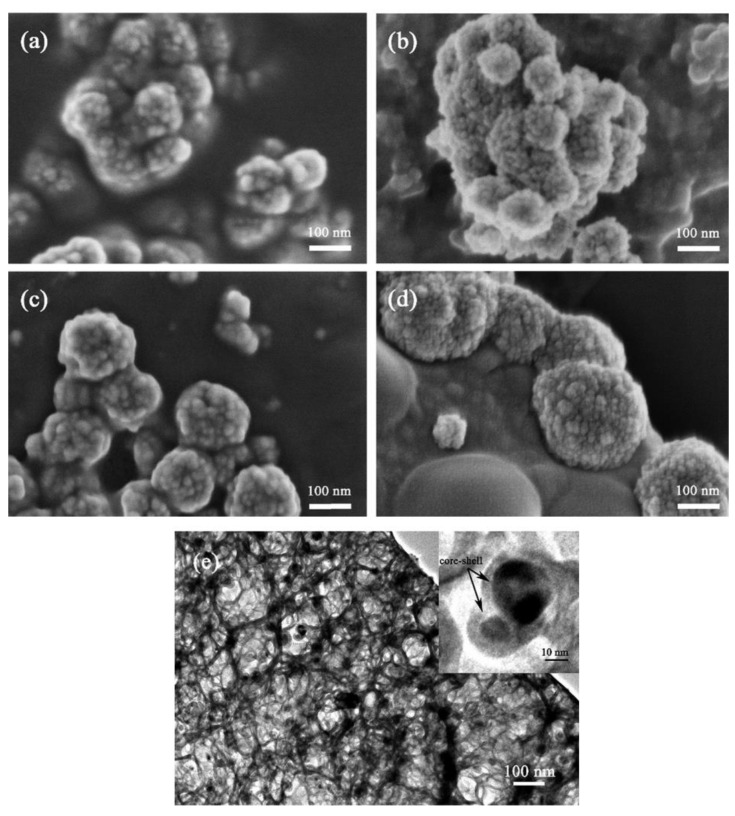
SEM images of (**a**) pure Cu_2_O, (**b**) GO/Cu_2_O-0.1, (**c**) GO/Cu_2_O-0.5, and (**d**) GO/Cu_2_O-1, and (**e**) a TEM image of GO/Cu_2_O-0.5 [64]. Copyright 2017, Elsevier.

Huang et al. [64] used the hydrothermal method to add graphene with the mass fractions of 0.1, 0.5, and 1 to Cu_2_O, which were noted as Cu_2_O/GO-0.1, Cu_2_O/GO-0.5, and Cu_2_O/GO-1, respectively (Figure 6). The experimental results showed that the highest hydrogen yield of Cu_2_O modified with graphene (118.3 mmol) was more than twice that of pure Cu_2_O (44.6 mmol). During the formation of Cu_2_O/GO composites, many negatively charged functional groups in graphene can recombine with positively charged copper ions by electrostatic adsorption, thus forming Cu_2_O/graphene composite structures directly during the reduction process. This principle has been used to synthesize cubic and octahedral Cu_2_O/GO composites. This structure could improve the efficiency of electron–hole separation. It could also improve the stability of the prepared catalysts. The experimental results showed that the cubic and octahedral Cu_2_O/GO composites degraded methyl orange with more than 90% efficiency. After six replicate tests, the efficiency remained above 70%, indicating that the prepared catalysts had excellent stability [65].

Graphene has properties such as the half-integer Hall effect, a unique quantum tunneling effect, and the bipolar electric field effect. In particular, its excellent electrical conductivity and huge specific surface area provide a feasible way to solve the bottleneck problem in the photocatalytic reaction of Cu_2_O-based composites.

### 3.2. Ternary Cu_2_O-Based Heterojunctions

In recent years, binary photocatalytic composites of Cu_2_O have achieved high achievements in the treatment of organic matter form wastewater and CO_2_ reduction. However, it will be a long time before binary photocatalytic composites can be used in society and daily life. Therefore, the development of ternary photocatalytic composites has become inevitable.

Yang et al. [66] used ternary Ag-CuO/GO as a photocatalytic material in the photocatalytic degradation of methyl orange, and the degradation efficiency of Ag-CuO/GO on the methyl orange was 90% after 60 min of visible light irradiation. Fu et al. [67] prepared TiO_2_-Ag-Cu_2_O composite catalysts for enhanced photocatalytic hydrogen production. The experimental results showed that the synergistic effect of Ag and Cu_2_O improved the photocatalytic efficiency of the reaction. In addition, the prepared composite catalysts had a double Z-scheme charge transfer pathway, which reduced the electron–hole complexation probability. The weak oxidation holes and weak reduction electrons in the charge transfer process were directly quenched, and the photogenerated carrier separation efficiency and catalyst reduction capacity were significantly enhanced.

### 3.3. Co-Catalyst Addition

In addition to constructing heterojunction structures, the photocatalytic efficiency can be improved by adding co-catalysts. Suitable co-catalysts are often present on the photocatalyst surface as active centers for oxidation or reduction, which can reduce the oxidation or reduction overpotential, and thus contribute to the photocatalytic reaction. In general, co-catalysts have three primary roles: (1) promoting the separation of the photoexcited electron–hole pairs; (2) inhibiting side reactions; and (3) improving the selectivity of the target products. Yu et al. [68] reported that adding the co-catalyst Cl to Cu_2_O nanorods led to a strong CO_2_ reduction ability. The experimental results showed that the addition of co-catalyst Cl mainly reduced its direct energy band, and also achieved an increase in the carrier density and conductivity. Zhang et al. [69] doped Zn in Cu_2_O microcubes, and the hydrogen production rate of Cu_2_O was six times higher than that of pure Cu_2_O when the Zn content was 0.1 wt.%. Kalubowila et al. [70] proposed a new method for introducing cocatalysts. They used ascorbic acid (AA) to reduce the prepared Cu_2_O/GO, where Cu_2_O was partially converted to Cu, and GO was fully converted to rGO. Cu nanoparticles with tens of nanometers have acted as co-catalysts in Cu_2_O/Cu/rGO composites, providing centers for effective charge transfer, and enhancing the performance of photocatalytic degradation.

## 4. Photocatalytic Applications

**Figure 7:**
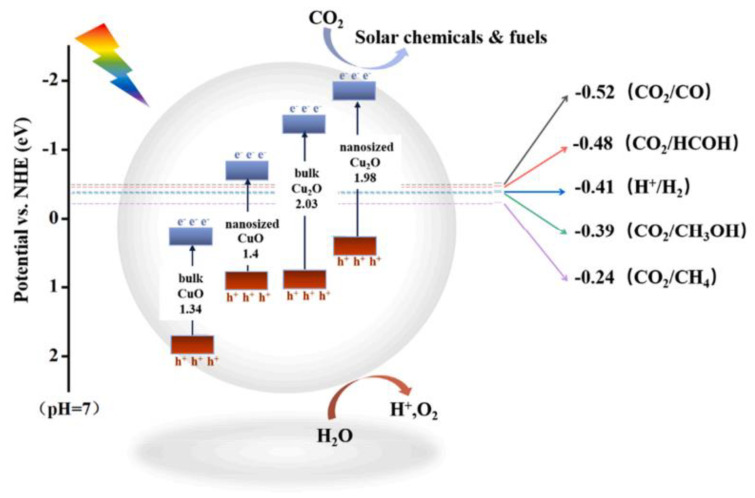
Diagram of the bandgap of copper-oxide-based photocatalysts [71]. Copyright 2022, Elsevier.

Semiconductor photocatalytic reactions are based on the solid energy band theory. Under the light, the available photogenerated electrons (*e^−^*) and holes (*h^+^*) in the conduction band (CB) and valence band (VB) of the semiconductor migrate to the surface, to participate in the redox reaction. Therefore, the appropriate match between the CB/VB position of the photocatalyst and the redox potential determines whether the reaction can occur. In general, the CB position of the photocatalyst should be more negative to the reduction potential of the reaction, to promote the transfer of *e^−^* from CB to the reactant; at the same time, the VB position should be corrected to the oxidation potential of the reaction, to ensure that holes can be transported from VB to the reactant. The bandgap of Cu_2_O-based materials is shown in Figure 7 [71]. It has been proven that they can be used as photocatalysts to achieve CO_2_ reduction (CO_2_RR), hydrogen production from water, pollutant degradation, and the reduction reaction of Cr. This section summarizes and discusses the latest progress in applications of Cu_2_O-based photocatalysts.

### 4.1. Photocatalytic CO_2_ Reduction

Photocatalytic technology can convert CO_2_ into CO and hydrocarbon fuels, achieving carbon recycling, and reducing greenhouse gas emissions. The application of Cu_2_O has been hampered largely by its inherent photocorrosion, ultra-fast charge recombination rate, and slow charge transport dynamics. In recent years, researchers have conducted and developed a series of novel Cu_2_O-based photocatalysts, making significant progress.

**Figure 8:**
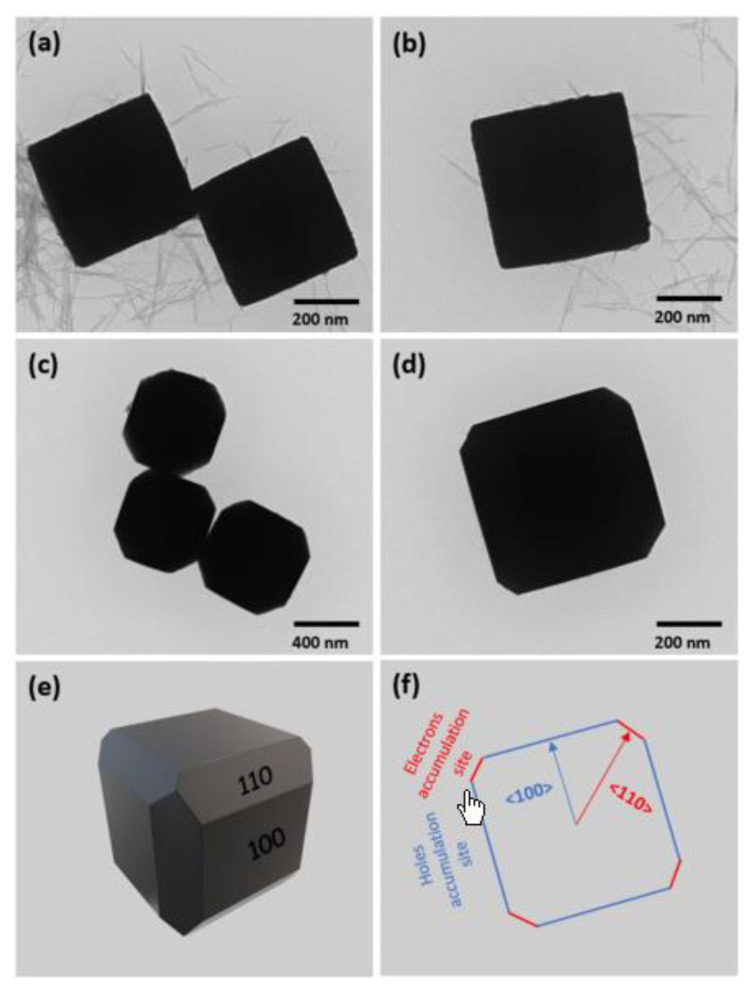
TEM images of (**a**,**b**) cubic Cu_2_O and (**c**,**d**) edge-truncated cubic Cu_2_O; simulated images of (**e**) 3D structure of edge-truncated cubic Cu_2_O and (**f**) 2D crystal orientation [74]. Copyright 2022 American Chemical Society.

As is well known, semiconductors with different morphologies often expose different crystal faces, and exhibit varying photocatalytic activity. Celaya et al. [72] calculated by density-functional theory (DFT) that the (110) and (111) crystal faces of Cu_2_O have the potential of photocatalytic reduction of CO_2_ to produce hydrocarbon derivatives. To further determine the catalytic mechanism and active site, Wu and his colleagues [73] successfully prepared Cu_2_O nanocrystals with (110) and (100) crystal faces through colloidal synthesis, and carried out photocatalytic reactions using CO_2_ and H_2_O. gas chromatography–mass spectrometry (GC-MS), confirming that methanol was the only product of photoreduction, and the internal quantum yield was approximately 72%. In photocatalytic reactions, the (110) surface of a single Cu_2_O particle showed photocatalytic activity, while the (100) surface was inert. The electronic density of the Cu active site on the (110) surface moved from Cu (i) to Cu (ii), and the oxidation state of the Cu changed from Cu (ii) to Cu (i) after CO_2_ conversion under light. In 2022, Sahu et al. [74] synthesized and characterized Cu_2_O photocatalysts with cubic and truncated cubic structures. Their correspondingly exposed crystal faces were different (Figure 8). Due to the selective accumulation of *e^−^* and *h^+^* on different crystal planes, the photocatalytic activity in selectively reducing CO_2_ to methanol on cubic Cu_2_O with anisotropic {100} and {110} crystal planes was nearly 5.5 times higher than that on cubic Cu_2_O with only {100} crystal planes.

Meanwhile, researchers have adopted various modification methods to optimize the structure and performance of the photocatalyst. Element doping is a commonly used method to effectively change the physical properties of semiconductors, to improve their catalytic activity. Cl doping has been shown to optimize the catalytic activity of Cu_2_O [75]. At 400 nm, the apparent quantum yield (AQE) of Cl-doped Cu_2_O photocatalytic reduction of CO_2_ to CO and CH_4_ increased, with 1.13% and 1.07% for CO and CH_4_, respectively. The reason behind the enhanced performance of CO_2_RR was not only that the Cl doping optimized the energy band structure and conductivity of Cu_2_O, and improved the adsorption capacity of CO_2_ and the separation efficiency of the photogenerated carriers, but also that the Cl-doped Cu_2_O was conducive to the conversion of CO_2_ into the intermediates of *COOH, *CO, and *CH_3_O, thus improving the yield and selectivity of CO and CH_4_.

Constructing heterojunction structures is also an effective method for band reconstruction. In heterostructures, the internal electric field is formed at the contact interface of two or more semiconductors with the movement of the Fermi level, which drives the directional migration and separation of photogenerated electrons and holes. Common heterojunctions include the traditional (Type-Ⅰ, Ⅱ, and Ⅲ), p–n, Z-scheme, and S-scheme. The p–n heterojunction of Cu_2_O and n-type semiconductors can effectively delay the recombination of photogenerated carriers, and promote electron transfer [76]. The yield of the photocatalytic reduction of CO_2_ to CH_3_OH from the Cu_2_O/TiO_2_ heterojunction after 6 h of UV–Vis irradiation has been 21.0–70.6 μmol/g_cat_. At the p–n heterojunction, the photogenerated electrons and holes are separated and transferred to the CB/VB with lower potential energy, respectively, resulting in a redox ability closer to the lower of the two semiconductors. The Z-scheme heterostructure solves this problem perfectly. Electrons and holes in the CB/VB with lower energy recombine, and cancel each other out in the Z-scheme heterojunction, thus retaining the higher conduction and valence band values in the two semiconductors, and enhancing the redox ability of the photocatalyst. For example, the Ag-Cu_2_O/ZnO nanorods (NRs) reported by Zhang and his team showed an enhanced photocatalytic CO_2_ reduction performance [77]. Under UV–vis light, the yield of CO significantly increased, which was seven times higher that of pure ZnO or Cu_2_O NRs. The results showed that the deposited Cu_2_O can enhance the chemical adsorption of CO_2_ on the catalyst surface, and the Z-scheme charge transfer pathway formed between the ZnO and Cu_2_O can promote effective charge separation, thereby improving the photocatalysis performance.

Due to the small bandgap energy and high conduction band value of Cu_2_O-based materials, the products of photocatalytic CO_2_RR are complex, mainly including CO and various organic compounds (CH_4_, CH_3_OH, HCOOH). According to the different reaction products, the application of Cu_2_O-based materials in photocatalytic CO_2_ reduction is summarized in Table 1.

### 4.2. Photocatalytic H_2_ Production

Hydrogen energy is abundant and renewable, which can effectively avoid energy exhaustion, and the products of hydrogen energy combustion will not cause pollution. Photocatalytic hydrogen production has the advantages of high efficiency, low cost, and environmental friendliness, and has great potential in high-efficiency hydrogen evolution. Common semiconductor photocatalysts (such as TiO_2_, ZnO, and g-C_3_N_4_.) have the disadvantage of a low utilization of sunlight, and the photocatalytic hydrogen evolution efficiency is not ideal. In recent years, Cu_2_O has become a research hotspot in photocatalytic hydrogen evolution because of its excellent photoresponsiveness. However, the poor charge separation ability of pure Cu_2_O lowers its hydrogen evolution performance. It is essential to modify and adjust Cu_2_O-based catalysts to meet the practical need to increase the hydrogen production yield.

**Figure 9:**
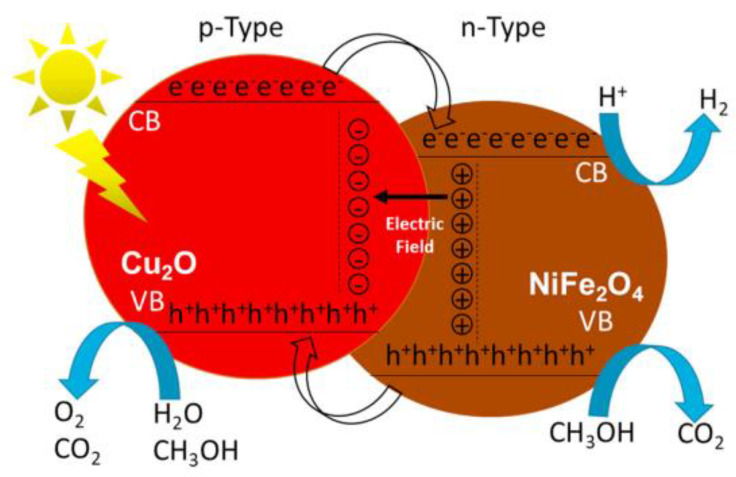
The p–n heterostructures in the NiFe_2_O_4_/Cu_2_O photocatalyst [94].

Hybridizing Cu_2_O with other semiconductor materials to construct heterojunctions can achieve the effective separation of photo-induced charge carriers, which is an effective method to enhance photocatalytic activity, and has been validated in numerous studies on photocatalytic hydrogen production. NiFe_2_O_4_/Cu_2_O with different mass percentages has been synthesized by impregnation and thermal annealing methods to construct p–n heterojunctions [94]. The photocatalytic hydrogen production rate of all heterojunctions was significantly higher than that of the original material. The 50/50 mass ratio was the most effective, and the hydrogen production rate within 24 h was 102.4 mmol∙g^−1^, while NiFe_2_O_4_ and Cu_2_O only obtained 1.35 and 0.85 mmol∙g^−1^, respectively. The increase in activity came from the enhanced charge separation at the heterojunction, which increased the concentration of charge carriers (Figure 9). Cu_2_O/CaTiO_3_ series samples were synthesized using the hydrothermal method and NaBH_4_ reduction treatment [95]. The photocatalytic hydrogen production effect of the 50Ca10Cu sample was the best (8.268 mmol∙g^−1^·h^−1^), about 344.5 times that of the CaTiO_3_ sample. It also exhibited perfect stability after multiple cyclic tests.

**Figure 10:**
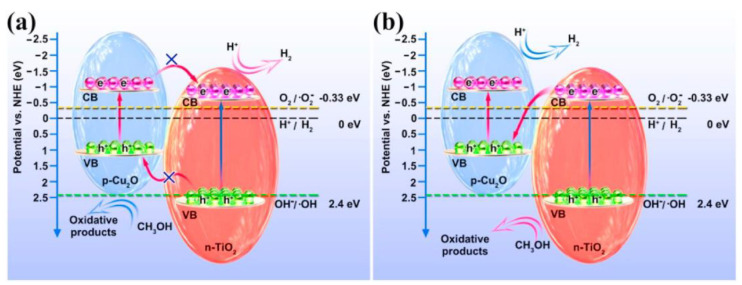
(**a**) Type-Ⅱ and (**b**) Z-scheme electron transfer mechanism in Cu_2_O/TiO_2_ photocatalyst [96]. Copyright 2021, Elsevier.

The above p–n heterojunctions are typical type-Ⅱ heterojunctions, which often impair the redox capacity of photogenerated electrons and holes. Researchers have recently designed and constructed Z-scheme and step-scheme (S-scheme) heterojunctions for photocatalytic hydrogen production. For example, dendritic branched Cu_2_O was synthesized hydrothermally, and Cu_2_O/TiO_2_ composites were prepared via surface charge modulation [96]. The hydrogen production rate of the optimized CT-70 (Cu_2_O coupled with 70 wt.% TiO_2_) photocatalyst reached 14.020 mmol^−1^ within six hours, which was 264 and 44 times higher than that of pure Cu_2_O and TiO_2_, respectively. The electron transfer mechanism of the Z-scheme was proposed and verified via DFT calculation and EPR analysis. Under simulated sunlight, photoexcited electrons migrate from the CB of TiO_2_ to the VB of Cu_2_O, and then recombine with photogenerated holes in the VB of Cu_2_O, thereby retaining highly reducing electrons and highly oxidizing holes (Figure 10). Therefore, under the conditions of sensitive photosensitivity and the effective separation of photogenerated electrons and holes, the performance of photocatalysts in hydrogen evolution under visible light is significantly improved. The S-scheme heterojunction photocatalyst has a similar efficient carrier separation performance and enhanced redox capacity. Cu_2_O/g-C_3_N_4_ composites were successfully synthesized using a simple wet chemical method, and applied in the field of photocatalytic energy production. Cu_2_O/g-C_3_N_4_ series samples showed high catalytic activity. In particular, 1-Cu_2_O/g-C_3_N_4_ showed the highest hydrogen evolution rate of 480.6 μmol∙g^−1^·h^−1^ under visible light irradiation, 12.0 times that of the original Cu_2_O sample. Based on the analysis of the experimental and simulation results, the ideal catalytic performance of the Cu_2_O/g-C_3_N_4_ photocatalyst was derived from the efficient interfacial charge separation and transfer of the S-scheme heterostructure [97].

Furthermore, photocorrosion is currently an urgent problem for Cu_2_O photocatalysts, and finding effective strategies to suppress photocorrosion in photocatalysts is still an enormous challenge. To overcome this challenge, Liu et al. [98] proposed a core–shell model: the Cu_2_O/PyTTA-TPA COF nanocube photocatalyst was constructed using an energy level matching the Cu_2_O and 2D PyTTA-TPA COF. It exhibited an excellent photocatalytic hydrogen evolution rate of 12.5 mmol∙g^−1^·h^−1^, approximately 8.0 and 20.0 times higher than the PyTTA TPA COF and Cu_2_O, respectively. Most importantly, under the protection of the stable PyTTATPA-COF shell, the Cu_2_O nanocube core was protected from photocorrosion, and did not show noticeable morphological or crystal structure changes after 1000 light excitations, thus significantly improving the photocorrosion resistance stability of the catalyst. Table 2 shows the recently reported Cu_2_O-based materials for photocatalytic hydrogen production.

### 4.3. Photocatalytic Degradation of Pollutants

With the rapid development of the global economy, industrial and agricultural waste is produced in large quantities, and continues to enter the environment. Many organic pollutants also enter the environment, and some show persistent pollution, which is difficult to remove through microbial action and hydrolysis. The long-term existence and accumulation of refractory pollutants leads to environmental pollution and ecological imbalance, and even threatens human survival and development. Research and development around pollutant degradation technology are critical. Photocatalytic technology has shown promising prospects for treating refractory pollutants, such as the photocatalytic processes that mineralize organic pollutants into water and CO_2_, and which essentially eliminate secondary pollution, rather than concentrating these pollutants and their by-products into the waste stream. In the past few decades, extensive research has been conducted on Cu_2_O-based photocatalysts to purify the environment. Table 3 summarizes the recent reports of Cu_2_O-based photocatalysts in pollutant degradation.

Among all the types of pollutants, organic dyes have become an important source of water pollution. As refractory organic pollutants, dyes cause severe damage to human health and the ecological balance. Traditional adsorption methods only transfer toxic organic molecules to the solid surface, without eliminating them, and still run the risk of desorption. MBC@Cu_2_O composites have been prepared by loading porous spherical Cu_2_O onto wood biochar carriers, with a liquid-phase synthesis strategy, at room temperature [109]. As a bi-functional adsorption-based photocatalytic composite, MBC@Cu_2_O showed great potential in removing anionic dye methyl orange (MO) from water. Under visible light irradiation, the photocatalytic degradation efficiency of MO reached 94.5%, and remained above 80% after five cycles. In another work, Sehrawat and his team prepared MoS_2_/Cu_2_O composites with different weight ratios via precipitation, using MoS_2_ nanosheets and Cu_2_O nanospheres [110]. The photocatalytic degradation of indigo carmine (IC) dye was carried out under simulated visible light. Compared to the original MoS_2_, the optimized MC-3 sample showed the best degradation performance, with a degradation rate of 99.59% for IC within 90 min, and no significant change in performance after five cycles. Experiments regarding the capture of active species showed that the photocatalytic reaction relied on the production of the superoxide radical (•O_2_^−^), and further verified the Z-scheme mechanism of the MoS_2_/Cu_2_O photocatalyst. In the same year, Li et al. synthesized the core–shell WO_3_-Cu_2_O Z-scheme heterojunction via hydrothermal and electrochemical deposition methods for the photocatalytic degradation of methylene blue (MB) under visible light [111]. The Cu_2_O nanoparticles deposited on the surface of WO_3_ enhanced the visible light absorption ability. The Z-scheme heterojunction achieved the effective spatial separation of the charges, and retained the strong redox ability of the photogenerated electrons and holes. The WO_3_-Cu_2_O-120s photocatalyst showed the highest reaction rate, almost twice that of the original WO_3_.

As a typical persistent organic pollutant, antibiotics are difficult to degrade and remove, due to their low biodegradability, which has become a thorny problem in water pollution control. Research has shown that the defect states and vacancies caused by element doping significantly impact the catalytic performance of semiconductor materials. Doping semiconductor functional materials with specific elements provides a feasible way to overcome the obstacles in applications for photocatalytic degradation. Nie et al. synthesized Cl-doped Cu_2_O microcrystals using a simple hydrothermal method, and used them to treat levofloxacin contaminants (LVX) under mild reaction conditions [112]. Compared with other reaction systems, the synthesis of Cl-doped Cu_2_O has a higher degradation efficiency for levofloxacin. After 240 min of photocatalytic reaction, the maximum degradation rate of LVX was 85.8% and 80.3% after eight cycles, indicating the stability and reusability of the photocatalyst. Based on the theoretical calculation and test results, it can be concluded that introducing hybrid orbitals and oxygen vacancy defects into Cu_2_O crystal cells by doping Cl reduces the band gap of Cu_2_O, resulting in a red shift in the absorption edge. Compared with pure Cu_2_O microcrystals, the prepared Cl-doped Cu_2_O single crystals with oxygen vacancy had a narrower band gap, and higher photogenerated electron–hole separation and transport efficiency. Considering the close relationship between the morphology and electronic structure, surface energy, and chemical reactivity of nanocrystals, it is of great significance to explore the influence of the morphology/exposed crystal surface of Cu_2_O on the synthesis process and the photocatalytic performance. Wu et al. developed a series of Cu_2_O@HKUST-1 core–shell structures via self-constrained strategies, using Cu_2_O nanocrystals with different morphologies as templates [113]. The characterization results indicated that the (111) surface of Cu_2_O was more favorable for the growth of HKUST-1 than the (100) surface. Comparing the photocatalytic degradation performance of tetracycline hydrochloride (TC-HCl), it was found that Cu_2_O@HKUST-1 had the best photocatalytic performance among the three types of composite material, with a degradation efficiency of 95.35% for TC-HCl. It was attributed to the excellent photoresponse, and the most effective interfacial charge transfer and separation in the Cu_2_O@HKUST-1 cubes.

In addition to organic dyes and antibiotics, solar-powered Cu_2_O-based photocatalysts can degrade heavy metal pollutants in wastewater, mainly toxic hexavalent chromium (Cr (Ⅵ)). Xiong et al. [114] constructed a Cu_2_O/LDH photocatalyst by grafting Cu_2_O-NP, and embedding it into the LDH host layer through an in-situ reduction strategy. CuZnTi LDH is valuable in two aspects: (a) as a source of Cu_2_O, and (b) as a support bracket to avoid the self-oxidation of Cu_2_O-NPs. The optimized photocatalyst showed a high degradation efficiency for difficult-to-degrade pollutants under visible light conditions, with a reduction rate of 95.5% for Cr (Ⅵ) by Cu_2_O/LDH0.10, and a degradation rate of 71.6% for TC. The excellent photocatalytic efficiency was attributed to the charge transfer mechanism of the Cu_2_O/ZnTiLDH p–n heterojunction, effectively promoting the separation and migration of the photogenerated electron–hole. Recently, Zhu et al. [115] used the Si and Cu of waste serpentine tailings and WPCB to prepare low-cost waste-based Cu-Cu_2_O/SiO_2_ photocatalysts. Due to the dispersion of Cu-Cu_2_O_3_ on the surface of the SiO_2_ carrier, the composite material obtained a higher specific surface area. The photocatalytic reduction of Cr (Ⅵ) using waste-based catalysts was the best at a loading rate of 9% Cu and 7g∙L^−1^ SiO_2_, and the photocatalytic activity decreased by only 4.93% after five cycles. The mechanism of Cr (Ⅵ) reduction by the waste Cu-Cu_2_O/SiO_2_ photocatalyst is to excite the waste Cu_2_O to produce photoelectron–hole pairs. The electrons in the waste group Cu_2_O CB reduce Cr (Ⅵ) adsorbed on the surface to Cr (Ⅲ), and the surface Cu drives the electrons to the surface of the Cu metal, without returning the waste group Cu_2_O.

Moreover, the accumulation in soil and water of herbicides, insecticides used in the agriculture and food industries, and phenolic compounds emitted from industry, such as petrochemicals and pharmaceuticals, can have significant harmful effects on humans and aquaculture systems. The use of metal oxide photocatalysts has been proven to be an effective, low-cost, and green method for treating such wastewater. In 2021, Alp [116] successfully synthesized hybrid Cu_2_O-Cu cubes by reducing D(+)-glucose in an alkaline solution using a one-step aqueous solution synthesis method, without any toxic reagents or surfactants. The Cu_2_O-Cu exhibited excellent photocatalytic properties for dyes and herbicides, due to the effective separation of photogenerated electron–holes and the enhanced charge transfer mechanism at heterojunctions. In particular, when dealing with 2,4-Dichlorophenoxyacetic acid (2,4-D), one of the widely used herbicides in agriculture and urban landscaping, the degradation effect of the Cu_2_O-Cu heterojunction was outstanding. It photodegraded all of the 2,4-D in the medium within 40 min, while the original Cu_2_O cube photodegraded 85% within 60 min. In the same year, Mkhalid et al. [117] prepared a Cu_2_O photocatalyst loaded with Cu nanoparticles via sol–gel and photo-assisted deposition technology. The structure and optical and photoelectric properties of the prepared photocatalyst were improved by adjusting the Cu content. The results showed that the band gap of the Cu_2_O loaded with 15% Cu was reduced to 1.95 eV, significantly enhancing the visible light absorption ability. The optimized Cu@Cu_2_O photocatalyst completely photodecomposed atrazine (AZ, a commonly used triazine herbicide) within 30 min, and demonstrated excellent durability. In recent years, effectively solving the problem of phenolic pollutants in livable environments has also been a major challenge faced by humanity, and has received a high level of attention from many researchers. A low-cost but highly efficient phosphate-doped carbon/Cu_2_O composite (HKUST-1-P-300) was reported by Dubai et al. [118]. The catalyst was derived from the modification of HKUST-1 with triphenylphosphine and conditioned calcination. Under visible light irradiation, the degradation efficiency of HKUST-1-P-300 for phenol was 99.8%, the hydrogen evolution rate was 1208 µmol, and the external quantum efficiency was 48.6% (at 425 nm) within 90 min, and the high performance could still be maintained after four cycles. Mechanism studies showed that the excellent photocatalytic activity of HKUST-1-P-300 came from multiple synergistic effects: an enhanced visible light absorption efficiency, a larger surface area, the effective separation of photogenerated carriers, a reduced aggregation of Cu_2_O, and the P-doped carbon/Cu_2_O structure. These novel Cu_2_O-based materials, as highly efficient photocatalysts, have potential applications in removing environmental pollutants, and generating clean energy, to promote sustainable environmental construction.

**Table 3:** Recently reported Cu_2_O-based materials for the photocatalytic removal of pollutants.

Photocatalyst	Synthesis Method	Morphology and Structure	Size	Bandgap (E_g_)	Light Resource	Targe Pollutant/Concentration/Volume	Efficiency	Cycle	Refs.
Ag-Cu_2_O	Electrochemical deposition and redox reaction	Composite film	—	2.02 eV	500 W halogen lamp	MB/30 mg∙L^−1^/50 mL	92%	3	[119]
Cr-doped Cu_2_O	Hydrothermal method	Octahedrons	800–1200 nm	2.06 eV	500 W tungsten halogen lamp (400–1100 nm)	LVX/40 mg∙L^−1^/50 mL	79.6–72.4%	1–8	[120]
BiOCl/Cu_2_O	Solvothermal method	Spherical shape	3–5 μm	2.00 eV	500 W Xenon lamp	Moxifloxacin/20 mg∙L^−1^/50 mL	72.3%	5	[121]
C-dots/Cu_2_O/SrTiO_3_	Hydrothermal and two-step method	Chocolate ball with sesame on the surface	~2.16 μm	Eg_SrTiO_3__: 3.19 eV; Eg_Cu_2_O_: 2.10 eV	500 W Xenon lamp (λ > 420 nm)	CTC.HCl/15 mg∙L^−1^/50 mL	92.6%	4	[122]
CuO-Cu_2_O	Chemical–thermal oxidation	Nanorods	60 nm	1.90 eV	150 W metal halide lamp (λ > 400 nm)	MB/5 mg∙L^−1^/50 mL	80%	3	[123]
Cotton fabrics/Cu_2_O-NC	Impregnation and HH reduction	Octahedron Cu_2_O attached to cotton fibers	20–40 nm of diameter of Cu_2_O	Eg_Cu_2_O_: 2.20 eV	350 W Xenon lamp (λ > 400 nm)	MB/200 ppm/200 mL	98.32–85%	1–5	[124]
Cu_2_O@HKUST-1	In-situ converted strategy	Octahedron structure	—	Eg_Cu_2_O_: 1.95 eV; Eg_HKUST-1_: 2.59 eV	Tungsten lamp (>420 nm, 500 W)	TC-HCl/20 mg∙L^−1^/100 mL	93.40–90.02%	1–4	[125]
Fe_3_O_4_/Cu_2_O-Ag	Solvothermal and liquid deposition methods	Double six peak structure	~5 nm	2.23 eV	—	PAHs/5 mg∙L^−1^/100 mL	95–90%	1–8	[126]
Cu_2_O/ZnO@PET	Electroless template deposition	Rectangular-shaped	~13 ± 4.5 nm	3.2–3.4 eV	Ultra-Vitalux 300W	Czm/1.0 mg∙L^−1^/100 mL	98–26%	1–6	[127]
Cu_2_O-Au-TiO_2_	Two-step photocatalytic deposition	Core–shell structure	~50 nm	1.4–1.7 eV	Xenon lamp (λ > 422 nm)	Cr(Ⅵ)/10 mg∙L^−1^/50 mL	100% (3h)	3	[128]
Cu_2_O/N-CQD/ZIF-8	Reduction precipitation	Spherical structure	~80–100 nm	2.6 eV,	300 W Xenon lamp (λ > 420 nm)	Cr(Ⅵ)/20 mg∙L^−1^/50 mL	98.99–97.13%	1–5	[129]
Cu_2_O/rGO/BiOBr	Two-step strategy	Hierarchical microspheres	500 nm–1 μm	Eg_BiOBr_: 2.7 eV; Eg_Cu_2_O_: 1.9 eV	300 W Xenon lamp (λ ≥ 420 nm)	Cr(Ⅵ)/20 mg∙L^−1^/50 mL	100% (40 min)	5	[130]
Cu-TiO_2_-Cu_2_O	Photodeposition	The triple junction structure	~20 nm	—	300 W Xenon lamp (200–2400 nm)	2,4,5-T/50 ppm/100 mL	93%	3	[131]
Ag-Cu_2_O/rGO	Two-step reduction process	Spherical AgNPs deposited on the Cu_2_O situated on the surface of rGO sheets	~60 nm	—	60 W tungsten filament lamp (500–700 nm, 0.24 W/cm^2^)	MO/40 mg∙L^−1^/50 mL Phenol/20 mg∙L^−1^/50 mL	90% (60min); Rate constant of phenol degradation: 0.09732	3	[132]

### 4.4. DFT Study Applied in the Photocatalysis

At the present time, there are fewer studies revealing the reaction mechanism of Cu_2_O through DFT simulations. Moreover, the catalytic microstructure and mechanism of Cu_2_O-based composites are still unclear. Designing Cu_2_O-based photocatalysts, and investigating the mechanism of improving photocatalytic activity at the molecular level require the introduction of theoretical calculations. In future studies, DFT simulations and experiments are needed, to reveal the relationship between the establishment of the microstructure and the catalytic activity of the photocatalysts, which will provide the theoretical basis for future photocatalytic industrial applications.

Lv et al. [133] analyzed the electronic structure and photocatalytic properties of Cu_2_O doped with different contents of Mn, using first-principle calculations. The simulation results showed that the visible light absorption intensity and photocatalytic efficiency were enhanced with the increase in doping concentration, and varied with the doping configuration, compared to pure Cu_2_O. The enhanced light absorption was mainly attributed to the in-band leaps of the electrons in the three-dimensional state of Mn. The enhancement of light absorption was mainly due to the in-band leaps of electrons in the three-dimensional state of Mn, which gave the semiconductor material certain metallic properties, and increased the absorbance of the visible light. Therefore, Cu_2_O applied to the future industrialization of photocatalysis could be doped with a small amount of Mn in the semiconductor, to improve the photocatalytic efficiency.

## 5. Conclusions

In recent years, the practical photocatalytic applications of Cu_2_O-based materials in scientific fields such as solar energy conversion and environmental remediation have attracted great interest. As a transition metal oxide, Cu_2_O has the advantages of a narrow band gap, strong visible light response, suitable conduction band position, low cost, and great potential as a photocatalyst. This paper introduces the basic properties, synthesis methods, and modification strategies of Cu_2_O-based materials. Recently reported Cu_2_O-based photocatalysts and their recent advances in photocatalysis, such as photocatalytic CO_2_ reduction, photocatalytic hydrogen production, and pollutant degradation, are reviewed. However, the research on Cu_2_O-based materials is still in its early stages, and there is room for improvement in their photocatalytic performance.

1. Currently, most Cu_2_O-based composites and sacrificial agents are synthesized from noble metal materials, which have high costs and significantly limit their large-scale applications. The development of non-precious metal catalysts, such as graphene, is vital to future development. More importantly, the catalytic efficiency of most Cu_2_O-based composites is very low, and the catalytic performance needs to be improved to meet the requirements of practical applications.
2. Although many experimental studies on the photocatalysis of Cu_2_O-based composites are introduced in this paper, these works are still in their infancy. In addition, the large-scale production of high-quality Cu_2_O-based photocatalysts faces numerous difficulties, considering the secondary hazards of nanomaterials. Therefore, it is urgent that we further study the photocatalytic mechanism of Cu_2_O-based composites from the above perspectives, and promote the industrial application process of Cu_2_O-based composite catalysts.
3. The photocorrosion of Cu_2_O still deserves attention. Although the current method of constructing heterojunctions to suppress photocorrosion has achieved certain results, the photocorrosion phenomenon of Cu_2_O still exists, and affects its long-term use. Establishing a core–shell structure is a good governance measure but, when synthesizing photocatalysts, it is necessary to carefully handle the thickness of the shell layer, to ensure sufficient absorption of light by the Cu_2_O.
4. The structure of the catalyst determines the catalytic activity, while the catalytic microstructure and mechanism of Cu_2_O-based composites is still unclear. Theoretical calculations should be introduced when designing a Cu_2_O-based photocatalyst, and studying the mechanism of improving photocatalytic activity at the molecular level. In future research, DFT simulations and experiments are needed, to reveal the relationship between the establishment of the microstructure and the catalytic activity of photocatalysts.

## Figures and Tables

**Table 1:** The application of Cu_2_O-based photocatalysts for CO_2_RR.

Photocatalyst	Synthesis Method	Morphology and Structure	Size	Bandgap (E_g_)	Light Resource	Product	Yield (μmol∙g^−1^ h^−1^)	Energy Conversion Efficiency/Selectivity	Refs.
Cu_2_O/Cu/CVO	Hydrothermal and wet chemical reduction methods	Cu_2_O nanoclusters and Cu NPs cover the surface of elliptic CVO NPs	~100 nm	Eg_CVO_: 2.34 eV; Eg_Cu_2_O_: 1.87 eV	300 W Xe lamp (λ > 400 nm)	CO and CH_4_	6.97 and 1.62	Selectivity: 51.3% for CO	[78]
3D porous Cu_2_O	Electrodeposition and thermal oxidation.	3D porous structure	23–25 μm	2.0 eV	300 W Xe lamp (λ > 420 nm)	CO, CH_4_, and C_2_H_4_	26.8, 4.04, and 0.66	—	[79]
Spherical Cu/Cu_2_O	Solution chemical method	Spherical structure	1 μm	—	300 W Xe lamp (λ > 420 nm)	CO, CH_3_OH, and H_2_	87.7, 10.2, and 5.4	—	[80]
Cu_2_O-Pd	AA reduction and in situ methods	Cube	~2 μm	1.90 eV	300 W Xe lamp (λ > 420 nm)	CO	0.13	—	[81]
Uio-66-NH_2_/Cu_2_O/Cu	Hydrothermal method	Octahedron UiO-66-NH_2_ and Cu attached to the surface of polyhedron Cu_2_O	1.5 μm	2.79 eV	300 W Xe lamp	CO	4.54	—	[82]
Cu_2_O-111-Cu^0^	One-pot method	Octahedral structure	side length of ~1 μm	1.98 eV	300 W Xe lamp	CH_4_	78.4	97%	[83]
Ag_4_/Cu_2_O@rGO	Water bath combining with gas-bubbling-assisted membrane reduction	Ultrathin rGO nanosheet and Ag NPs supported on Cu_2_O octahedral nanocrystals	Cu_2_O: 300 nm; Ag: 10.7 nm; rGO: 1.0 nm (thickness)	Eg_Ag/Cu_2_O_: 1.92 eV; Eg_Cu_2_O_: 2.0 eV	300 W Xe lamp (λ > 380 nm)	CH_4_	82.6	AQE: 1.26%. Selectivity: 95.4%	[84]
1D Cu_2_O@Cu NRs	In situ reduction method	One-dimensional nanorod arrays	<100 nm	2.03 eV	350 W Xe lamp (λ > 420 nm)	CH_4_ and C_2_H_4_	—	AQE: 2.4%	[85]
RT-Cu_0.75_	Low temperature thermochemical reduction and photo-deposition	—	—	2.72 eV	100 W solar simulator with an AM 1.5 filter	CH_4_	77 nmol·g^−1^ h^−1^	AQE: 0.012%	[86]
U-Cu_2_O-LTH@PCN-X	In situ reduction	Ultrafine nanoclusters	<3 nm	Eg_PCN_: 2.62 eV; Eg_U-Cu_2_O-LTH_: 2.07 eV	300 W Xe lamp (λ > 400 nm)	CH_3_OH	51.22	AQE: 1.01%	[87]
Fe_3_O_4_@N-C/Cu_2_O	AA reduction and aerobic oxidation	Rod-shaped core–shell nanostructure	5 nm (thickness of NC shell layer)	—	5 W Xe HID lamp	CH_3_OH	146.7	—	[88]
Dodeca-Cu_2_O/rGO	Solution-chemistry	Rhombic dodecahedra	400–700 nm	2.16 eV	300 W Xe lamp (λ > 420 nm)	CH_3_OH	17.765	—	[89]
Carbon layer@CQDs/Cu_2_O	Hydrothermal method	Nearly spherical structure	~2 µm diameter	2.09 eV	300 W Xe lamp	CH_3_OH	99.6	—	[90]
Ti_3_C_2_ QDs/Cu_2_O NWs/Cu	Self-assembly strategy	QDs incorporated onto NWs	~500 nm (diameter of NWs)	2.02 eV	AM 1.5, 300 W Xe lamp	CH_3_OH	78.50	—	[91]
Cu@Cu_2_O	Thermal treatment	Core–shell nanoparticles	~70 nm diameter	—	Xe lamp (420–780 nm)	HCOOH	67.35	AQE: 0.12% at 560 nm	[92]
NH_2_-C@Cu_2_O	Low temperature annealing	Octahedral structure	—	1.79 eV	300 W Xe lamp (λ > 420 nm)	HCOOH	138.65	Selectivity: 92%	[93]

**Table 2:** The reported applications of Cu_2_O-based materials in photocatalytic hydrogen production in recent years.

Photocatalyst	Synthesis Method	Morphology and Structure	Size	Bandgap (E_g_)	Light Resource	Yield (μmol∙g^−1^ h^−1^)	Energy Conversion Efficiency/Selectivity	Refs.
Cu_2_O/TiO_2_	Ball-milling	Irregular shapes	Anatase: 16.2 nm Rutile: 30.5 nm	3.08 eV	High-pressure Hg lamp (125 W)	200	AQE: 1.51% Light-to-chemical energy efficiency: 0.6%	[99]
In(OH)_3_-In_2_S_3_-Cu_2_O	Hydrothermal, wet chemical and electrospinning process	Nanofiber	100–200 nm of diameter	Eg_In(OH)_3__: 5.15 eV Eg_In2S3_: 1.98 eV Eg_Cu_2_O_: 2.17 eV	5 W blue light LED (λ_max_ = 420 nm)	1786.5	—	[100]
Au@Cu_2_O	Sequential ion-exchange reaction	Core–shell architectures	54.4 ± 4.8 nm	Eg_Cu_2_O_: 2.40 eV	Xenon lamp and AM 1.5G filter	55.5	AQE: 0.29% at 420 nm	[101]
Na_2_Ti_6_O_13_/CuO/Cu_2_O	Solid-state and impregnation method	Belt morphology	1 μm	3.61 eV	UV/vis lamp (254 nm, 4400 μW/cm^2^)	33	—	[102]
C@Cu_2_O/CuO	Calcination	Chrysanthemum-like crystalline	—	2.0 eV	350 W Xe lamp (40 mW/cm^2^)	26,700	External quantum efficiency (EQE): 52.4%	[103]
NiCo-LDH/Cu_2_O	Electrostatic self-assembly	3D flower cluster	—	Eg_NiCo-LDH_: 1.78 eV Eg_Cu_2_O_: 1.89 eV	5 W LED (λ ≥ 420 nm)	3666	—	[104]
Cu_2_O/TiO_2_	DES-assisted synthesis	Cu_2_O nanoclusters on TiO_2_ surfaces	1.5 nm of Cu_2_O nanoclusters and 25.8 nm of TiO_2_ particles	Eg_TiO_2__: 3.12 eV Eg_Cu_2_O_: 2.13 eV	300 W Xe lamp	24,210	—	[105]
Cu@TiO_2_-Cu_2_O	Hydrothermal and NaBH_4_ treatment	Urchin-like hierarchical spheres	—	Eg_TiO_2__: 3.18 eV Eg_Cu_2_O_: 2.05 eV	300 W Xenon lamp	12,000.6	AQE: 8.26%	[106]
Cu/Cu_2_O	Microwave-assisted heating	Hollow spherical morphology	430 ± 1.2 nm in diameter	2.0 eV	LED light (20 W)	141	—	[107]
Cu_2_O/SiO_2_/CdIF	Reactive deposition	Core–shell structure	—	Eg_CdIF_: 5.09 eV Eg_Cu_2_O_: 2.22 eV	300 W xenon lamp (340–780 nm)	2879.09	AQE: 0.040% at 420 nm	[108]

## Data Availability

Not applicable.

## Sample Availability

Samples of the compounds are available from the authors.
